# Comparative analysis of the USA’s Washington Ferries and road transport carbon emissions using the Trozzi and Vaccaro and Greatest Integer functions

**DOI:** 10.1007/s11356-023-28281-7

**Published:** 2023-06-28

**Authors:** Kadir Mersin, Metin Yıldırım, Andrew Adewale Alola

**Affiliations:** 1 Logistics Management, Faculty of Economics, Administrative and Social Sciences, Istanbul Gelisim University, Istanbul, Turkey; 2 CREDS-Centre for Research on Digitalization and Sustainability, Inland Norway University of Applied Sciences, 2418 Elverum, Norway; 3 Faculty of Economics, Administrative and Social Sciences, Nisantasi University, Istanbul, Turkey

**Keywords:** Environmental sustainability, Road and waterways, Trozzi and Vaccaro function, Greatest Integer function, United States

## Abstract

Countries’ sectors are currently under great scrutiny for their response to the greenhouse gas (GHG) emission profile and the general effect of the sectoral activities on the environment. As in the agenda of all sectors, environmental concerns and investigations are of high importance in shipping and maritime transport. Amidst the rising forms of globalization, the need for sustainable transportation is constantly increasing. However, the machines that are the cornerstone of transportation largely depend on fossil fuels, thus resulting in environmental degradation. Notably, environmental-related degradation has continued to account for global warming, climate change, and ocean acidification. Shipping is considered the most environmentally friendly mode of transportation in terms of carbon dioxide (CO_2_) emissions per ton per mile of transported unit load when compared against road transportation. In this study, six ferry lines (FLs) of Washington State Ferries were calculated to compare ship-generated carbon dioxide (CO_2_) emissions with those from road transportation as if the carried vehicles had used the highway instead of transport by FL. While making these calculations, the Greatest Integer function (GIF) and Trozzi and Vaccaro function (TVF) were utilized. From the examined three scenarios, i.e., all passengers travel by car instead of ferry as scenario 1, all ferries carry both cars and passengers as scenario 2, and all car-free passengers travel by bus instead of ferry as scenario 3, the outlined results are as follows: (i) none of the cars were carried by the ferry, and car-free passengers preferred traveling by their own cars as observed in scenario 1; (ii) hypothetical scenarios (1 to 3) in which the road vehicles carried on FLs had instead used the highway, and the total potential CO_2_ emissions of these road vehicles were calculated as 2,638,858.138, 704,958.2998, and 1,394,148.577 tonnes per year, respectively. Policy-wise, this study revealed the management strategies for CO_2_ emissions reduction for two transport modes, shipping and road transportation, under current conditions.

## Introduction

With the provision or access to mobility of humans, goods, and services, transportation is arguably the most essential connector. By recognizing the impact of transportation on fostering inclusive growth through creating more access to essential services and mitigating the growing global effect of climate change, intergovernmental agencies are not willing to relent in aiding the partnership of government and private institutions (World Bank, 2021a). However, there remain many challenges to achieving a sustainable global transportation system. For instance, the World Bank (2021a) reported that one billion people still live more than 2 km from accessible road transportation systems, thus causing about one and half million deaths annually. Moreover, in spite of the growing benefits and contributions of the global transportation system, there are other far-reaching problems associated with the current model. Arguably, the climate change-related issues associated with the global transportation system are increasingly worrisome. For instance, the global contribution of greenhouse gas emissions from transportation amounts to 16% of the global GHG emissions amidst a $15 billion annual cost of direct damages to the transportation infrastructure from natural disasters (World Bank, 2021a). Specifically, in the world’s largest economy, the United States, the energy sources mainly used for transport come from the burning of fossil fuels (mostly petrol, gasoline, and diesel); therefore, the sector contributed 29% of the country’s 2019 total greenhouse gas emissions (United States Environmental Protection Agency, 2021).

Importantly, global warming, which accounts for the increase in average temperatures of land, sea, and air throughout the year, has continued to pose a serious challenge to the global transportation system. The reason is that the transportation networks across land, sea, and air are directly or indirectly impacted by the increase in the greenhouse gases (GHGs), i.e., CO_2_, H_2_O (water, which could be in the form of vapor), and CH_4_ (methane, which is an organic compound), with the CO_2_ emissions being the dominant contributor to global warming (Winnes et al., 2015). The global average surface temperature increased between 0.6 and 0.9 °C from 1906 to 2005, and the rate of temperature increase has almost doubled during this time range, especially within the last 50 years. According to a report from The National Oceanic and Atmospheric Administration’s (NOAA) National Centres for Environmental Information (NCEI), nine of the world’s 10 hottest summers have occurred since 2005 (NOAA, 2020). Moreover, five of these 10 summers have occurred since 2015. In 2019, the average temperature worldwide was 0.95 °C higher than the average temperature of the twentieth century and is only 0.04 °C lower than the highest recorded temperatures of 2016 (NOAA, 2020). With the COVID-19 pandemic that emerged in 2019, a decrease in (GHG) emissions has occurred that is largely associated with a slump in transportation logistics (Magazzino et al., 2021; World Bank, 2021b). By early June 2020, global daily fossil fuel CO_2_ emissions were mostly back to 5% below 2019 levels (UN Environment Programme, 2020).

As identified in the literature, fossil fuels are mainly responsible for the increase of GHGs (Alola, 2019; Ike et al., 2020; Usman et al., 2020; Koyuncu et al., 2021; Onifade et al., 2021). Notably, vehicle-based CO_2_ emissions are the largest factor driving the increase, which is especially the case for the United States transport sector (Umar et al., 2021). Therefore, the current study considers the case of the United States transportation sector because of its significant contribution to the country’s greenhouse gas emission profile (United States Environmental Protection Agency, 2021). Specifically, this study considers the ferries and road transport systems in the state of Washington, whose transportation sector is the largest contributor to the state’s total greenhouse gas emissions, amounting to about 99.6 million metric tons in 2018 (The Seattle Times, 2021; Reducing greenhouse gases, 2021). This study employs a novel approach for estimating the CO_2_ emissions from vehicles, thereby providing a comparative analysis of the United States’ Washington Ferries and road transport systems. By using the Trozzi and Vaccaro function (TVF) and Greatest Integer functions (GIF), this study offers a new approach for the ship-based CO_2_ emissions and is compared with the tier method that is employed for road vehicles. By so doing, the current study expectedly contributes to the existing literature, thus narrowing the gap in the transport-environmental sustainability literature.

Additional background information highlighted with relevant literature is provided in the “Background: sectoral emissions” section. In the “Data collection and methods” section, the dataset and empirical methods employed are presented in a specified order. While the results of the study are highlighted in the “Results of the scenarios” section, the results are discussed in the “Discussion of results” section. The summary of the study with relevant policy suggestions is offered in the “Conclusion and policy implication” section.

## Background: sectoral emissions

Beginning with the use of carbon-based fuels in transportation, an increase in anthropogenic greenhouse gas emissions has been observed. As of 2019, greenhouse gas emissions were 6558 million metric tons of carbon dioxide equivalents in the United States. Of that value, 29% was produced by the transportation sector. This value caused the transportation sector to appear at the top of the greenhouse gas emission producer sectors list in the United States (Environmental Protection Agency, 2021). According to 2019 figures from the United States, more than two-thirds of human-caused greenhouse gas emissions were CO_2_ emissions. The origin of 74% of GHG emissions and 92% of total anthropogenic CO_2_ emissions is associated with the utilization of carbon-based fuels in the United States (Energy Information Administration (EIA), 2021). It is observed that the rate of anthropogenic GHG emissions increased in the time period between 2000 and 2010 and is much higher than the rate of increase seen in the previous three decades. As of 2010, anthropogenic greenhouse gas emissions reached 49 gigatons CO_2_ per year, which was the highest figure in human history (Intergovernmental Panel on Climate Change, 2021). As of 2017, greenhouse gas emissions in the European Union were 4.483 megatons of CO_2_e, which was 21.7% lower than 1990 values (European Environment Agency, 2019, 2021).

It is observed that studies on environmentally sustainable transportation strategies aiming to reduce greenhouse gas emissions are increasing. One approach proven to yield a decrease in GHG emissions is the use of renewable energy sources. The use of renewable energy in the transportation sector has been revealed as one of the reasons behind the increase in real disposable personal income, also causing a decrease in CO_2_ emissions (Alola & Yildirim, 2019; Shafiei & Salim, 2014). Environmentally sustainable transportation has been defined by The Organization of Economic Cooperation and Development (OECD) as “Transportation that does not endanger public health or ecosystems and meets mobility needs consistent with (a) use of renewable resources at below their rates of regeneration and (b) use of non-renewable resources at below the rates of development of renewable substitutes” (OECD, 1997).

### Empirical literature highlight

Bouman et al. (2017) reviewed over 150 research papers related to GHG emissions reduction for the maritime transport sector. The authors concluded that GHG emissions could be reduced by a factor of 4–6 per freight unit transported by only using the currently available technologies (Bouman et al., 2017). It has been observed that the use of automatic mooring systems at RoRo/Pax ports causes a decrease in CO_2_ emissions, and it is predicted that a 97% reduction can be achieved at RoRo/Pax ports (Díaz-Ruiz-Navamuel et al., 2018). Bridges over the straits are basically an alternative to short-sea shipping. Bridges over Dardanelle resulted in higher CO_2_ emissions than those caused by the transit ships passing through Dardanelle (Mersin, 2020). When evaluating external costs of air emission, Lee et al. (2010) estimated that the use of short-sea transport instead of truck transportation is one of the most important alternatives for reducing external costs.

Additionally, Svindland and Hjelle (2019) demonstrated that increasing the ship size would not cause an increase in CO_2_ efficiency in every case of short-sea transport. Christodoulou and Cullinane (2020) showed evidence of the environmental benefits of short-sea shipping. The technical and operational initiatives of shipping companies have been associated with the source of these benefits (Christodoulou & Cullinane, 2020). In a study conducted on ferry services to the islands, Baird and Pedersen (2013) indicated that CO_2_ emissions would decrease if shorter sea routes were preferred over longer sea routes. However, another study on the comparison of approximately 900 short-sea shipping and road lines demonstrated that short-sea transport is not always more environmentally friendly than road transport, contrary to the popular generalization (Kotowska, 2015).

When comparing the result of CO_2_ emissions from short-sea and road transportation in the Marmara region, Ülker et al. (2020) revealed that a CO_2_ emission comparison should be conducted for each line and region before any conclusions can be made. Hjelle and Fridell (2012) showed in their study that whether Ro-Ro transport achieved lower CO_2_ emissions compared to those of road transport mainly depended on the market situation that is attained with load factors. Given the result of Hjelle and Fridell (2012) analysis, it is clear that the use of both low-sulfur fuel and the latest abatement technologies have not been sufficient for Ro-Ro transportation. As such, the study revealed that the outcome is not capable of providing superiority in environmental performance compared to road transport and that Ro-Ro ships should cruise well below their design speeds (Hjelle, 2011). Meanwhile, a study of the impact of both biomass energy and fossil fuel energy utilization in the entire transportation sector in the United States revealed interesting results (Umar et al., 2021). Notably, Umar et al. (2021) revealed that biomass energy utilization in the transport sector mitigates CO_2_ emissions, while the utilization of fossil energy sources is responsible for a surge in CO_2_ emissions.

In the present study, environmental performance with regard to CO_2_ emissions of Washington State Ferry lines has been examined and compared with those of road transport systems. Given the uncommon direction employed when compared to existing literature, the study potentially makes a significant contribution to the body of knowledge available on the topic.

## Data collection and methods

In this part of the study, the adopted method for estimating carbon emissions from transportation via Washington Ferries and road transportation are detailed with discussion of the results. However, we begin the description with the related dataset that is employed for the investigation.

### Data collection

**Fig. 1:**
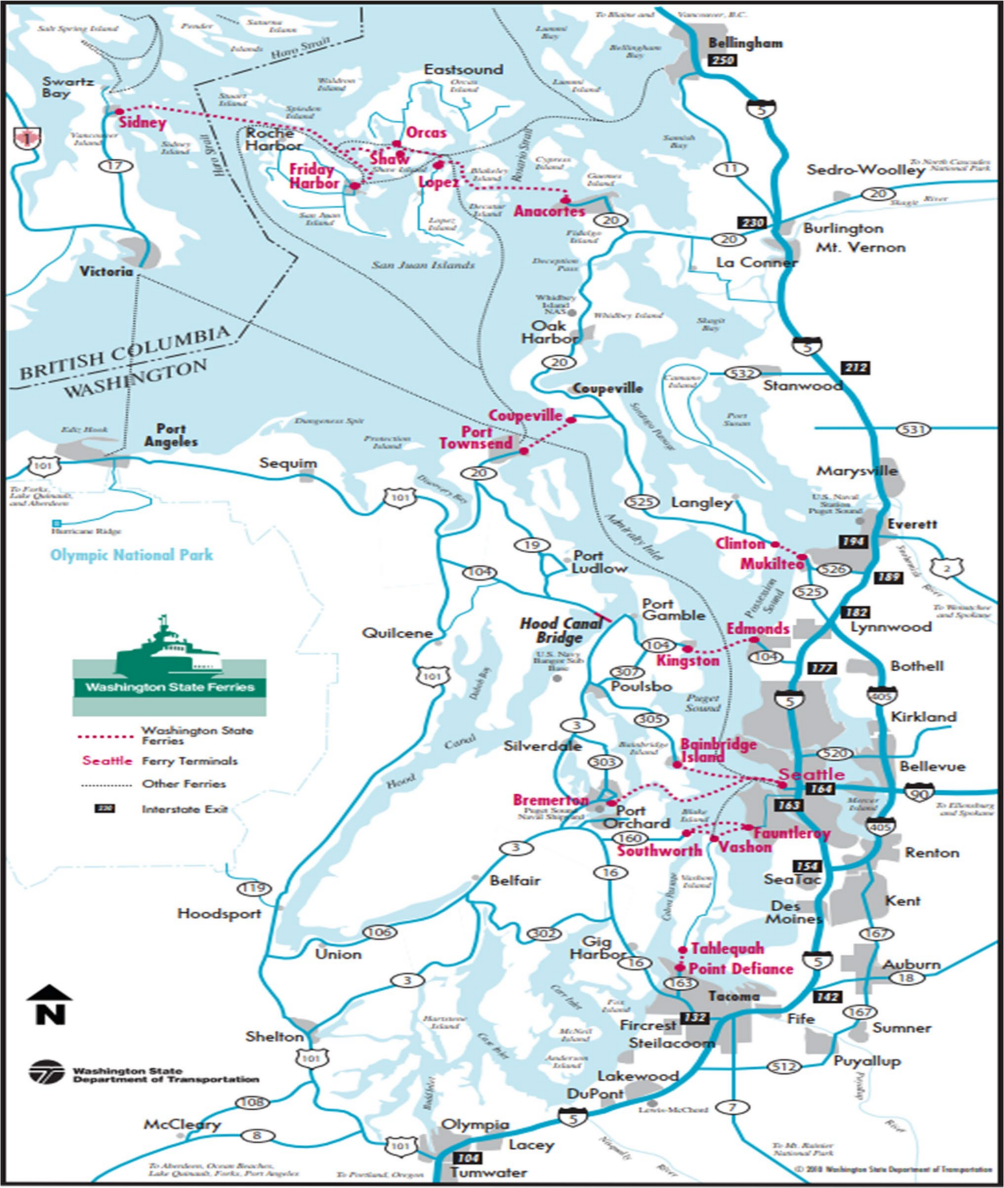
Washington State Ferry lines

The Washington State Department of Transportation (2021a) website issues information about ferry lines and live ferry status. Frequency of the ferry lines are also shown on the website. A map of the Washington State Ferry lines is demonstrated in Fig. 1. Car capacity, passenger capacity, speed, and displacement of the vessels can be seen by visiting the above website, and the implemented information is outlined in Table 1 (Washington State Department of Transportation, 2021b).

**Table 1:** Properties of Washington State Ferry vessels

Name	Displacement (tonne)	Speed (kt)	Car capacity	Passenger capacity	Route	Number of trips per year
Puyallup	6184	18	202	2500	Edmonds-Kingston	9464
Tacoma	6184	18	202	2500	Seattle-Bainbridge Island	8528
Walla Walla	4860	18	188	2000	Edmonds-Kingston	9100
Kaleetan	3634	17	144	2000	Seattle-Bremerton	6968
Yakima	3634	17	144	2000	Anacortes-San Juan Islands	7300
Chimacum	4384	17	144	1500	Seattle–Bremerton	5096
Samish	4384	17	144	1500	Anacortes–San Juan Islands	5475
Suquamish	4384	17	144	1500	Mukilteo–Clinton	5475
Tokitae	4384	17	144	1500	Mukilteo–Clinton	13104
Cathlamet	3310	16	124	1200	Fauntleroy-Vashon Island-Southworth	4004
Kitsap	3310	16	124	1200	Southworth-Fauntleroy-Vashon	14560
Sealth	3310	16	90	1200	Fauntleroy-Vashon-Southworth	5564
Kittitas	3310	16	124	1200	Mukilteo-Clinton	8528
Kennewick	2415	15.5	64	750	Port Townsend–Coupeville	7280
Salish	2415	15.5	64	750	Port Townsend–Coupeville	4368

According to the Washington State Department of Transportation website, some vessels are not listed in the schedule. Therefore, these vessels are not included in the calculations for this study

### Trozzi and Vaccaro method

A method for computing the total emissions of pollutants was introduced by and named after Trozzi and Vaccaro (1999). One advantage of this approach is its efficient use for the quantification of emissions and diffusion of pollutants that emanate from different environmental scenarios. The formula employed for the estimation method of CO_2_ emissions is given in Eq. (1).

1 $$E\left(t_\mathrm{total}\right)=\sum\nolimits_{i=1}^3\mathrm{C}\times f\times t\times p_i$$

where

*E*(*t*_total_): the total amount of CO_2_ emissions for *t*-day sailing

*C*: fuel consumption (tonne)

*f* = 3200 kg/tonne (CO_2_ emission factor)

*t*: time (day)

*p*_1_: sailing mode multiplier (0.8)

*p*_2_: maneuvering mode multiplier (0.4)

*p*_3_: hotelling mode multiplier (0.2)

According to the above formula, three different situations should be examined while making calculations: sailing, port, and maneuvering. In this study, port and maneuvering emissions are neglected because the time variable is taken as days in the calculations. If port time and maneuvering time are converted to days, there will be little change in the calculations. Thus, these changes can be neglected.

#### Trozzi and Vaccaro function

Fuel consumption is the largest contributor to CO_2_ emissions. There are some formulas for fuel consumption that neglect weight of the ship or changes in weight. By employing the approach from most recent studies, a new formula was built that does not neglect instant weight changing and shows the displacement tonnage at any time *t* (Mersin et al., 2017). Thus, we imply that $\nabla (t)={\left(\sqrt[3]{\nabla (0)}-\frac{\lambda {v}^3t}{3}\right)}^3$ and fuel consumption for *t* day is $C(t)=\nabla (0)-{\left(\sqrt[3]{\nabla (0)}-\frac{\lambda {v}^3t}{3}\right)}^3$, where ∇ is displacement of the ship, in other words, weight of the ship. *v* is speed of the vessel, ∇(0) is the initial displacement of the ship (at time *t* = 0), and *λ* is a parameter that is approximately equal to 1/120,000 for diesel machinery installation. Therefore, this formula can be written as $\nabla (0)-{\left(\sqrt[3]{\nabla (0)}-\frac{v^3t}{8640000}\right)}^3$ for the hourly emission.

In this part of the study, a function is built by modifying the Trozzi and Vaccaro method. Accordingly, there is an emission factor for each exhaust gas such that the CO_2_ emission factor is 3.2 tonnes per tonnes. Additionally, given the function modification, the mode multiplier is 0.8 for sailing such that daily emission can be computed by multiplying daily fuel consumption with emission factor for CO_2_ and mode multiplier. In light of the above facts, for this study, the modified function from the TVF is expressed in Eq. (2) as

2 $$\mathrm{TVF}(t)=\left[\nabla (0)-{\left(\sqrt[3]{\nabla (0)}-\frac{v^3t}{8640000}\right)}^3\right]\times 3.20\ x\ 0.80=\left[\nabla (0)-{\left(\sqrt[3]{\nabla (0)}-\frac{v^3t}{8640000}\right)}^3\right]\times 2.56$$

#### Calculation of potential CO_2_ emissions of road vehicles (tier 1 method)

Tier emission calculation methods are divided into various levels according to their activity and technological features. Tier 1 is a method that requires less data compared to the tier 2 or tier 3 methods (Ülker et al., 2020). The tier 1 method can be used for CO_2_ emission calculation if detailed features of the vehicle are not known. In this method, emission factors are based on fuel consumption for different vehicle types. The factors are given in the EMEP/EEA Guidebook (2019). Accordingly, the formula of the tier 1 method is given in Eq. (3).

3 $${E}_i={\sum}_j\left({\sum}_m\Big({FC}_{j,m}\times {EF}_{i,j,m}\right)$$

*E*_*i*_: emission of *i* pollutant (g)

*FC*_*j*, *m*_: fuel consumption for the vehicle that is in category *j* and using fuel *m* (kg)

*EF*_*i*, *j*, *m*_: emission from the pollutant *i* for vehicle category *j* and fuel *m* (g/kg)

In the United States, diesel-powered vehicles account for 4% of all motor vehicles (Chambers & Schmitt, 2015). Therefore, only gasoline cars and diesel-powered buses are considered in this study. Characteristic fuel consumption of cars and buses are assumed 70 g/km and 100 g/km, respectively, such that *EF*_*i*, *j*, *m*_ is taken as 3.18 grams per gram of fuel consumption.

In light of the above facts, the tier 1 method can be modified as

4 $$E\left(\mathrm{car}\right)=3.18\times 70\times d=222.6\times d$$

5 $$E\left(\mathrm{bus}\right)=3.18\times 100\times d=318\times d$$

where *E*(car) and *E*(bus) are CO_2_ emissions of a car and a bus, respectively. *d* is distance between two points. In this study, distances are acquired from https://www.rome2rio.com/map/.

**Fig. 2:**
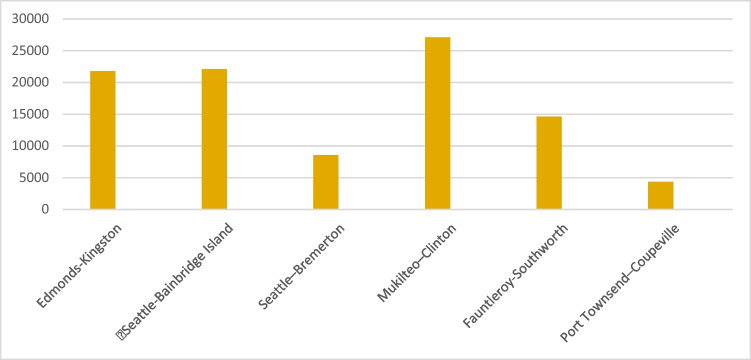
Annual ferry emissions (measured CO_2_ emissions, tonne)

According to the function in Eq. (2), annual CO_2_ emission of the ferries can be calculated. Thus, the annual emissions of ferry lines are given in Table 2 and graphically illustrated in Fig. 2.

**Table 2:** Annual ferry emission

Route	CO_2_ emissions (tonne)
Edmonds-Kingston	21,799.1011
Seattle-Bainbridge Island	22,086.3667
Seattle-Bremerton	8563.53482
Mukilteo-Clinton	27,101.9346
Fauntleroy-Southworth	14,632.8255
Port Townsend-Coupeville	4337.48604

## Results of the scenarios

In scenario 1, it is assumed that all passengers travel by car instead of ferry and that each car has four passengers. In this case, the number of cars can be calculated by the Greatest Integer function (GIF). For all real numbers *x*, the Greatest Integer function returns the largest integer less than or equal to *x*. In essence, it rounds down a real number to the nearest integer. For example, GIF (3, 4) = 3, GIF (5) = 5.

According to Table 1, the number of cars that will be on the road can be calculated by Eq. (6), and results of the calculation are given in Table 3.

6 $$\mathrm{NC}\mathrm{R}=\mathrm{GIF}\left(\mathrm{NP}-4\ast \mathrm{NC}/4\right)+1.$$

**Table 3:** Number of cars that will be on the road for each trip

Route	Distance (km)	Number of car
Edmonds-Kingston	168.1	736
Seattle-Bainbridge Island	148.2	780
Seattle-Bremerton	104.5	355
Mukilteo-Clinton	169.2	408
Fauntleroy-Southworth	95.4	563
Port Townsend-Coupeville	339	124

where

NCR: number of cars that will be on the road

NP: number of passengers that are carried

NC: number of cars that are carried

**Fig. 3:**
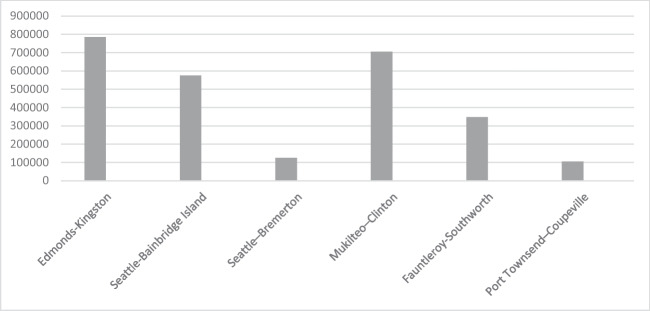
Annual car emissions (measured CO_2_ emissions, tonne)

According to Eq. (4), the potential annual CO_2_ emissions from cars are given in Table 4 and Fig. 3. Of course, it is assumed that all cars have the same properties. For example, all cars have the same fuel consumption rate and are gasoline cars.

**Table 4:** Annual car emission

Route	CO_2_ emissions (tonne)
Edmonds-Kingston	785,430.121
Seattle-Bainbridge Island	575,614.018
Seattle-Bremerton	123,943.734
Mukilteo-Clinton	704,347.846
Fauntleroy-Southworth	346,242.929
Port Townsend-Coupeville	103,279.49

In scenario 2, all ferries carry both cars and passengers. In other words, ferries carry car-free passengers as well. In this scenario, it is assumed that all car-free passengers travel by bus instead of ferry and that each bus has 51 passengers. These assumptions are conceived from the information provided by the maritime activities (available at https://wsdot.wa.gov/ferries) in the examined cases. In this case, the number of buses that will be on the road is attained with the help of the GIF in Eq. (7) and outlined in Table 5.

**Table 5:** Number of buses that will be on the road for each trip

Route	Cars	Passengers	Number of buses
Edmonds-Kingston	390	4500	58
Seattle-Bainbridge Island	346	4500	62
Seattle-Bremerton	208	2250	28
Mukilteo-Clinton	268	2700	32
Fauntleroy-Southworth	338	3600	45
Port Townsend-Coupeville	64	750	10

NBR = GIF((NP − 4*NB)/51) + 1.(7)

where

NCR: number of buses that will be on the road

NP: number of passengers that are carried

NB: number of buses that are carried

**Fig. 4:**
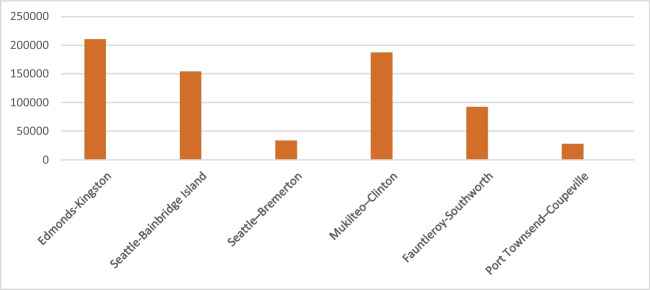
Annual bus emissions (measured CO_2_ emissions, tonne)

Produced from Eq. (5), the potential annual CO_2_ emissions of buses is given in Table 6 and Fig. 4. Of course, it is assumed that all buses have the same properties. For example, all buses have the same fuel consumption rate and that all of them are diesel cars; this is an important assumption utilized in the computations.

**Table 6:** Annual bus emissions

Route	CO_2_ emissions (tonne)
Edmonds-Kingston	210,172.02
Seattle-Bainbridge Island	154,027.657
Seattle-Bremerton	33,538.5611
Mukilteo-Clinton	186,952.852
Fauntleroy-Southworth	92,369.842
Port Townsend-Coupeville	27,897.3677

**Fig. 5:**
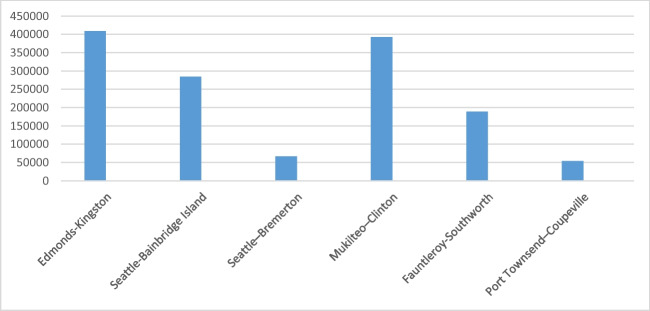
Total annual emissions (measured CO_2_ emissions, tonne) of scenario 3

In scenario 3, it is assumed that all car-free passengers travel by bus instead of ferry and that each bus has 51 passengers. Furthermore, none of the cars are carried by ferry. For example, according to Table 5, 390 cars and 58 buses are carried per trip in Edmonds-Kingston Ferry line. In scenario 3, passengers preferred traveling by their own car or by bus instead of ferry. Of course, scenario 3 operates on the same assumption as scenario 1 and scenario 2, i.e., that the cars/buses exhibit the same energy consumption rates and utilize the same energy sources. The total annual emissions of this scenario (3) are given in Table 7 and Fig. 5.

**Table 7:** Total annual emissions of scenario 3

Route	CO_2_ emissions (tonne)
Edmonds-Kingston	409,006.664
Seattle-Bainbridge Island	284,176.231
Seattle-Bremerton	66,659.3736
Mukilteo-Clinton	392,115.688
Fauntleroy-Southworth	188,433.4
Port Townsend-Coupeville	53,757.2206

## Discussion of results

**Fig. 6:**
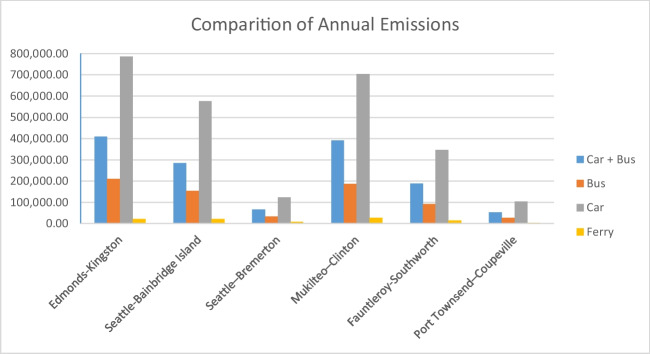
Comparison of annual emissions (measured CO_2_ emissions, tonne)

The existing literature on the subject of the study shows the necessity of comparing CO_2_ emission values on the basis of ferry lines. In parallel with the studies in the existing literature, this study includes six different Washington State Ferry lines and provides a separate evaluation made for each line. Three possible basic alternatives have been identified in the use of road transportation, but the first two alternatives consist of sole usage of private and public transportation vehicles, namely, cars and buses. The last alternative consists of usage of cars and buses at the same time. Comparison between relevant alternatives has been conducted on full load assumption of ferries. Three scenarios are created, and each of them are compared with emissions caused by ferries, which is shown with the yellow row in Fig. 6.

According to scenario 1, none of the cars are carried by the ferry, and car-free passengers preferred traveling with their own cars. Annual emissions caused by scenario 1 are shown with the gray row in Fig. 6. Scenario 2 occurs when all passengers preferred traveling by buses. The annual emissions caused by scenario 2 are shown with the orange row in Fig. 6. Finally, scenario 3 is the case when none of the cars are carried by the ferry, and car-free passengers preferred traveling by buses. The annual emissions caused by scenario 3 are shown with the blue row in Fig. 6.

In the hypothetical scenarios 1, 2, and 3 where the road vehicles carried on FLs had used the highway, the total potential CO_2_ emissions of these road vehicles are calculated as 2,638,858.138, 704,958.2998, and 1,394,148.577 tonnes per year, respectively. The values observed from the investigation point led to a series of important conclusions and recommendations. The first observed point is that the CO_2_ emission ranking of the transportation options is the same for each of the six ferry lines. Studies from the literature have emphasized that in CO_2_ emission comparisons, differences might be detected on the basis of lines and that it is necessary to perform separate analysis for each line (Baird & Pedersen, 2013; Cooper, 2001; Pizzol, 2019; Ančić et al., 2020). For instance, Baird and Pedersen (2013) found a significant difference in the CO_2_ emissions between the long-range ferry services (using the route from Aberdeen to Orkney Islands) and the short-range ferry services (across the Pentland Firth). Although the short-range ferry services consisted of a longer road journey for vehicles than the long-range ferry services, findings revealed that there are greater CO_2_ emissions for the long-range ferry services. Additionally, Cooper (2001) compared the pollutant emissions from three different on-board high-speed passenger ferries that are all marine diesel engines and characterized with the following: the first one uses conventional, medium-speed; the second one is a gas turbine engine; and the third one is a conventional, medium-speed equipped with selective catalytic reduction (SCR) systems for NOx abatemen. The result revealed that the second ship showed lower emissions of NOx, PM, and PAH but not without higher fuel utilization and CO_2_ emissions. However, in the current study, the relevant situation was not determined. The results of the investigation yield the same conclusion for all the investigated ferry lines. Another important point revealed by the results of the analysis is that maritime transport causes the lowest CO_2_ emissions on the relevant lines among other options. It should be noted that the calculations have been conducted on the maximum capacity for ferries. The figures show that higher CO_2_ emission values will be endured if different road transport alternatives are preferred over ferries for the investigated lines. This situation supports the previous studies, which state the importance of high load factor requirements at short-sea transportation in order to achieve lower CO_2_ emissions when compared to alternative transportation modes (Jebli & Belloumi, 2017; Ülker et al., 2020; Dujmović et al., 2022). Specifically, the investigation by Ülker et al. (2020) showed interesting results when comparison is made between CO_2_ emissions from short-sea shipping and road transportation in a Turkish region. The investigation showed that the total emission budget of the examined Ro-Ro and ferry lines is higher than the potential CO_2_ emissions of the vehicles being carried. Thus, the study established that carbon emissions can be minimized by shifting trucks from the highway to the seaway. Meanwhile, among the other three scenarios in which highway alternatives are examined, the bus-only scenario has the lowest CO_2_ emissions.

## Conclusion and policy implication

Looking at the existing literature about the current study, there is a necessity to compare the CO_2_ emission values on ferry line basis with road transportation. The case with the United States is crucial due to the economic prowess and carbon emission profile of the country. Considering this motivation, the current study examines six different Washington State Ferry lines (because of their importance in the country) by using a separate evaluation for each ferry line. Additionally, three possible basic alternatives were identified in the use of road transportation, where the first two alternatives consist of sole usage of private and public transportation vehicles, namely, cars and buses. The last alternative consists of usage of cars and buses at the same time. Importantly, the result revealed that road vehicles carried on FLs had instead used the highway such that the total potential CO_2_ emissions of these road vehicles are respectively computed as 2,638,858.138, 704,958.2998, and 1,394,148.577 tonnes per year. Comparison between relevant alternatives was carried out on full load assumption of ferries on one hand and road transportation on the other. In a novel approach, the study adopted the Trozzi and Vaccaro function and Greatest Integer function; as such, the results offer relevant policy implications with notable research limitations and recommendations.

### Policy and recommendations for future study

Considering that the state of Washington produces a desirable amount of carbon-free electricity that potentially meets about 90% of its demand (Washington Policy Center, 2020), a sector-specific legislation that is aimed at achieving such desirable success could be formulated to prevent transport emissions. Additionally, to complement the state’s more recent transport-related emission regulations (such as the Clean Car Law, Zero Emission Vehicle (ZEV) standard, and the Clean Fuel Standard), more partnership with the private sector could offer significant energy and climate finance, such as the Volkswagen Federal and State settlements (Reducing greenhouse gases, 2021). Moreover, in order to consistently reduce carbon emissions in both maritime and land transportation modes, there should be incentives for sea and land users to optimize the shortest possible routes.

The current study deals with carbon emissions in maritime and land transportation; other greenhouse gases and/or pollutant emissions could be considered in a similar research framework. Moreover, provided there is no data limitation, the study could be extended to other states and/or other economies.

## Data Availability

Data are available upon request from the corresponding author.
